# Habitat Selection of the Smooth‐Coated Otter ( *Lutrogale perspicillata* ) in Shuklaphanta National Park, Nepal

**DOI:** 10.1002/ece3.71297

**Published:** 2025-05-26

**Authors:** Balram Awasthi, Suraj Baral, Babu Ram Banjade, Grace M. Yoxon, Purna Man Shrestha

**Affiliations:** ^1^ Department of Zoology, Siddhanath Science Campus Tribhuvan University Mahendranagar Nepal; ^2^ Biodiversity Research and Conservation Society Kathmandu Nepal; ^3^ LIB, Museum Koenig Leibniz Institute for the Analysis of Biodiversity Change Bonn Germany; ^4^ Nepal Zoological Society Kathmandu Nepal; ^5^ International Otter Survival Fund Broadford UK; ^6^ Wildlife Research and Education Network Kathmandu Nepal; ^7^ IUCN Species Survival Commission, Otter Specialist Group Gland Switzerland

**Keywords:** habitat selection, human disturbances, smooth‐coated otters, vegetation

## Abstract

Smooth‐coated otters are top predators, semi‐aquatic carnivores, and keystone species in the aquatic ecosystem, serving as important biological indicators of wetland health. The species, once widespread across the Terai, is now limited to buffer zones and protected areas. This research investigated the habitat selection of smooth‐coated otters within the Shuklaphanta wetland landscape, an ecologically significant area in the Western Terai lowland of Nepal. The field survey was conducted using line transects with a length of 200 m in a 600 m long section along the bank of the Chaudhar River and the wetlands. A total of 71 line transects were sampled, 53 in the Chaudhar River and 18 in the wetlands. Of these, 15 transects in the Chaudhar River and 18 in the wetlands revealed signs of the presence of smooth‐coated otters. Five habitat variables (canopy cover, water channel width, river bank slope, bank substrate, and human disturbance) play important roles in the presence of otters. The relationship between otter presence and habitat variables was determined using binomial logistic regression. The probability of smooth‐coated otter presence increased with an increase in canopy cover, a higher proportion of sand and mud, and wider water channels. In contrast, otter presence decreased with an increase in the Human Disturbance Index and bank slope. However, average depth and water current appeared to have no significant effect on the presence of smooth‐coated otters. Regular monitoring of smooth‐coated otter habitats and vegetation, along with reducing anthropogenic activities, is urgently needed to conserve the smooth‐coated otter and its habitat in the western lowlands of Nepal.

## Graphical abstract

Smooth‐coated otters, important indicators of wetland health, are now limited to protected areas in Nepal. This study examined their habitat selection in Shuklaphanta National Park, finding that canopy cover, water channel width, and substrate type positively influence their presence, while human disturbance and bank slope negatively impact it. Regular habitat monitoring and reducing human activities are recommended for their conservation.

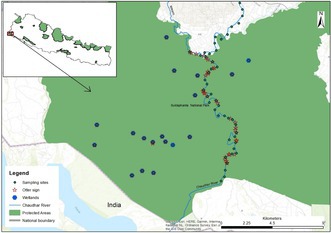

## 1 Introduction

Out of 13 otter species recorded worldwide, three species—Eurasian otter ( *Lutra lutra* ), Asian Small‐clawed otter ( *Aonyx cinereus* ), and Smooth‐coated otter ( *Lutrogale perspicillata* )—are currently present in Nepal (Acharya and Rajbhandari 2011; Shrestha et al. 2025; Acharya et al. 2023). The Asian small‐clawed otter, however, has only just been rediscovered in the country in 2025, which was first recorded in 1839 (Hodgson 1839; Shrestha et al. 2025). The smooth‐coated otter was once widespread in wetlands both inside and outside the protected areas of the Terai region of Nepal (Acharya 1998; Acharya and Lamsal 2010; Acharya and Rajbhandari 2012), but its distribution is currently restricted to small patches in buffer zones and protected areas of Koshi Tappu Wildlife Reserve, Chitwan, Bardia, and Shuklaphanta National Parks (Mishra et al. 2022; Acharya and Lamsal 2010; Gwachha et al. 2023; Bhandari 2019; Acharya 2006, 2017; Thapa et al. 2021). Furthermore, a continuous decline has been documented for smooth‐coated otters in Nepal (Acharya and Lamsal 2010; Acharya and Rajbhandari 2011; Jnawali et al. 2011; Thapa et al. 2021). The illegal trade in otters and increasing human disturbances, such as infrastructure development and pollution, have reduced otter populations and their natural habitats in Nepal (Acharya 2017; Acharya and Rajbhandari 2011; Savage and Shrestha 2018).

Smooth‐coated otters, as semi‐aquatic carnivores and keystone species in freshwater ecosystems, play vital roles in maintaining ecological balance and serve as indicators of wetland health (Kruuk 2006; Khan et al. 2014). They prefer shallow waters with moderate currents, soft riverbanks, and riparian vegetation with dense canopy cover (Raha and Hussain 2016; Weinberger et al. 2019).

Populations of smooth‐coated otters have significantly declined due to factors such as water quality degradation, overfishing, poaching for fur, habitat destruction, human disturbance, and disruptions caused by hydropower plants (Moser et al. 1996; de Silva et al. 2015), which have negatively impacted their distribution (Acharya and Rajbhandari 2014; Thapa et al. 2021). Classified as Vulnerable in the IUCN Red List (Khoo et al. 2021; IUCN 2024) and listed in Appendix I of CITES (CITES 2021), the species is not prioritized under Nepal's National Park and Wildlife Conservation Act, 1993. However, a 2002 amendment to the Aquatic Life Protection Act (Government of Nepal 2017), prohibits the hunting and killing of smooth‐coated otters nationwide (Savage and Shrestha 2018).

Habitat selection is a hierarchical process that influences various behaviors, leading to a non‐random use of available habitat (Yoxon and Yoxon 1990; Jonah Dias et al. 2022). Assessing habitat selection provides valuable insights into the behavioral responses of organisms and their distribution across habitats of varying ecological quality (Morrison et al. 1992; Arlt 2007; Gwachha et al. 2023). For smooth‐coated otters, specific habitat features, including bank slope, river width, river depth, resting sites, grooming sites, and breeding sites, are critical to their occurrence and overall ecology (Mason and Macdonald 1986; Madsen and Prang 2001; Melisch et al. 1996; Anoop and Hussain 2004; Nawab and Hussain 2012a, 2012b; Raha and Hussain 2016). The otters avoid areas lacking adequate resting sites and escape cover, even when food availability or water quality is favorable (Raha and Hussain 2016; Basak et al. 2021). Understanding these features and otter distribution is essential for informing conservation strategies and ensuring the long‐term persistence of otter populations in their natural habitats (Acharya et al. 2023).

Smooth‐coated otters are the most extensively studied of the Asian otter species, with substantial research on their behavior, diet, and ecology (Hussain 1996, 2002; Basnet et al. 2020; Acharya et al. 2023). These studies have provided valuable insights into their dietary preferences, social behavior, and ecological roles (Anoop and Hussain 2004; Nawab and Hussain 2012a; Raha and Hussain 2016; Basak et al. 2021). Habitat selection in Nepal has been studied in parts of Chitwan and Bardia National Parks (Acharya and Lamsal 2010; Gwachha et al. 2023). However, their habitat selection, particularly in specific regions, remains less explored. This study aims to investigate the habitat preferences of smooth‐coated otters in Shuklaphanta National Park (ShNP), to enhance our understanding of their ecological needs and support future conservation efforts. The study evaluates how environmental factors influence otter presence, with a focus on canopy cover, substrate type, water channel width, bank slope, and human disturbances. The hypotheses are as follows: There is a negative association between human disturbance—measured through the Human Disturbance Index—and the presence of smooth‐coated otters; There is a positive association between the presence of smooth‐coated otters and habitat features such as wider water channels, greater canopy cover, and finer substrate (sand and mud); There is a negative association between otter presence and steeper bank slopes, which may reduce suitable habitat availability.

### 1.1 Study Area

**FIGURE 1:**
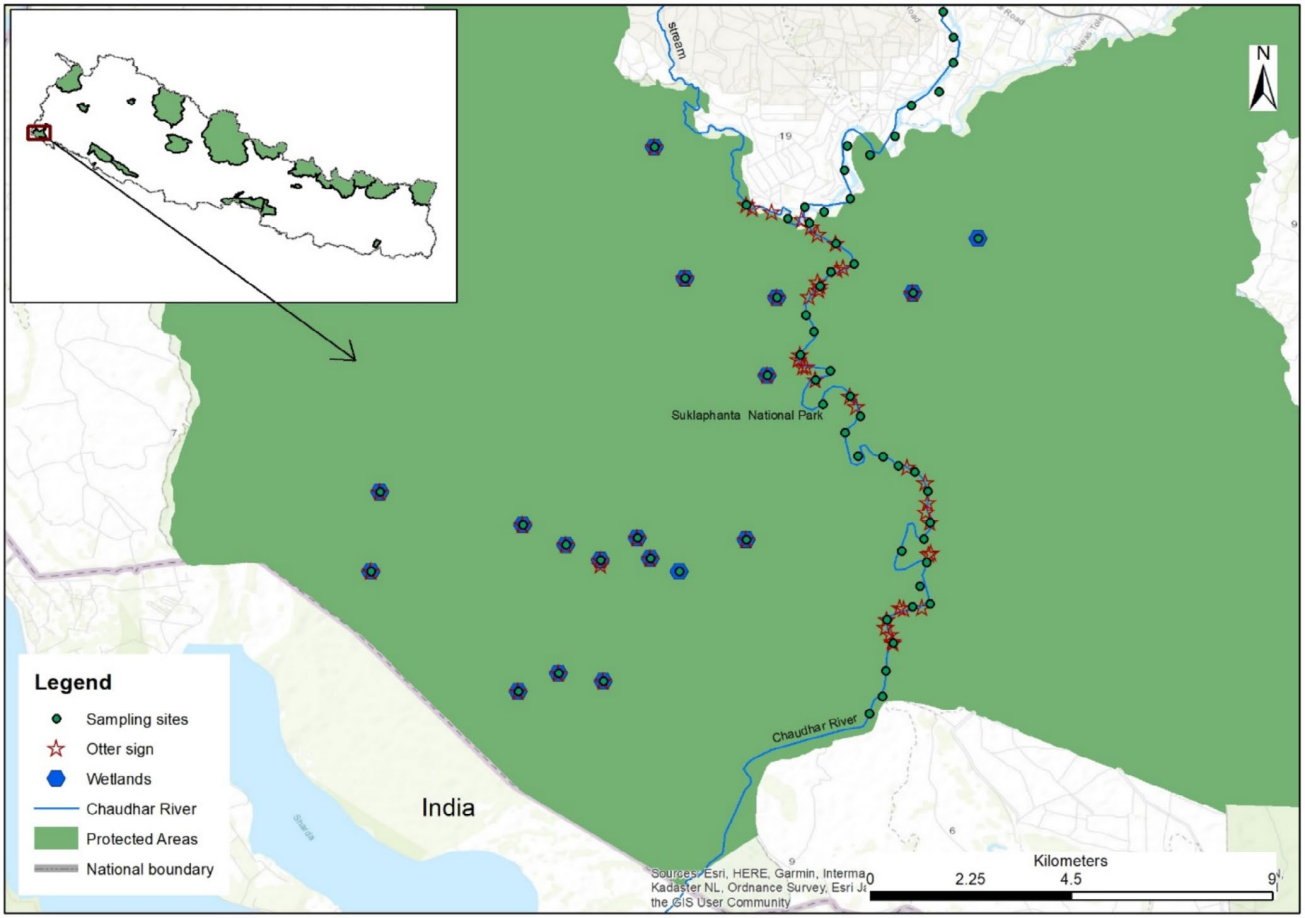
Map of the study area showing the sampled area in Shuklaphanta National Park.

Shuklaphanta National Park is located in the southwest of Nepal (Figure 1) and covers an area of 305km^2^, bounded by the Syali River in the east, the Mahakali River in the west, the Siwalik Hills in the northeast, and the Pilibhit Tiger Reserve and Dudhwa Tiger Reserve in the southeast of India (Poudyal and Chaudhary 2019; Department of National Park and Wildlife Conservation 2003). The climate is subtropical monsoonal, with three distinct seasons: cool‐dry (late September to mid‐February), hot‐dry (mid‐February to mid‐June), and monsoon (mid‐June to late September) (Poudyal et al. 2021). ShNP supports a wide range of biodiversity, including 665 floral species, 15 amphibian species, 56 reptile species, 456 bird species, 57 species of mammals, and 24 fish species (Rawat et al. 2020). There are mixed habitats of grasslands, wetlands, and mixed forests, forming a mosaic of wildlife habitats (Rawat et al. 2020). The study area includes the Chaudhar River, a key waterway that flows through the Park. This river, along with the surrounding wetlands, is a vital habitat for smooth‐coated otters, providing the necessary conditions for their survival (Thapa et al. 2021; Joshi et al. 2021). The river's varying features—such as water quality, vegetation, and proximity to human settlements—play an important role in shaping the otter's habitat preferences (Awasthi et al. 2024). This area is crucial for understanding otter distribution and their ecological needs within the park.

## 2 Methods

The field survey was conducted from October 2020 to March 2021 in the Chaudhar River and surrounding wetlands of ShNP. The sampling sites in the wetlands and rivers were identified through a preliminary survey visiting locations inside the protected area and through consultation with Park officials. A sampling method of line transects with a length of 200 m and a width of 10 m in each 600 m segment was carried out along the banks and shorelines of the rivers and wetlands. The length of the Chaudhar River from the southern border of the protected area to the base of the Churia hills, was divided into a number of segments 600 m long. Basak et al. (2021) surveyed three transects, 250 m in length within 1 km, and surveyed them twice. So, in order to best align with that sampling method, we surveyed 200 m in 600 m long sections. Along each transect, a search was conducted for otter signs within 10 m perpendicular to the shoreline to indicate presence/absence (Kruuk and Conroy 1987; Hussain and Choudhury 1995; Anoop and Hussain 2004; Basak et al. 2021). Both direct signs (sightings and calls) and indirect signs (tracks, scat, grooming sites, latrine sites, and dens) were sampled and recorded on data sheets. GPS coordinates were recorded for the presence or absence of smooth‐coated otters along the line transects using a handheld Global Positioning System receiver (Garmin etrex 10).

In addition to the presence and absence of the species, various habitat variables were measured in the surveyed plots: water depth of river and wetlands measured by a scale on a stick just 1 m from the bank; water channel width (shortest distance between banks of the river or wetlands measured by a 50 m measuring tape); water current (flow of water measured through visual observation); distance of shoreline vegetation from the water's edge measured by a measuring tape; bank substrate calculated based on visual observation; and the number of fallen trees counted by visual observation (Table 1). Additionally, overall substrate types like the proportion of sand/mud, small stones, big stones, and boulders in streams/rivers and lakes were recorded, along with the shoreline of each site (Table 1). Substrate attributes were categorized by diameter as follows: 1) sand (< 5 mm), 2) pebbles (5 mm‐5 cm), 3) small stones (5‐50 cm), 4) large stones (50‐100 cm), and 5) boulders (> 100 cm) (Jamwal et al. 2016; Shrestha et al. 2021) (Note that the total percentage may not equal 100% due to the use of mid‐points in calculations). Canopy cover was estimated by percent cover class using a Densiometer, categorized as 0%–25% (lightly vegetated), 25%–50% (moderately vegetated), and 50%–100% (heavily vegetated) (Russavage et al. 2021). The water current, bank substrate, and human disturbance were measured by visual observation.

**TABLE 1:** Predictor variables.

Variable	Description	Measure	Type of variable	Assumption/justification
Flow	Water flow velocity	Slow	Categorical	Surface water/visually estimated
		Fast		
		Stagnant		
Slope	Bank slope	Degree	Numerical	Visually estimated
Bank condition	Condition of bank	High disturbance	Categorical	Visually estimated
		Moderate disturbance		
		No disturbance		
Anthropogenic activities	Disturbance factor	Livestock grazing	Binary	Presence/absence
		Illegal sand mining	Binary	Presence/absence
		Fishing	Binary	Presence/absence
		Solid waste(pollution)	Binary	Presence/absence
		Human disturbances	Binary	Presence/absence
		Infrastructure development	Binary	Presence/absence
		Washing and bathing	Binary	Presence/absence
Human Disturbance Index^a^	HDI	Low disturbance	Categorical	Visually estimated
		High disturbance		
Fallen tree	Numerical	Number of fallen trees	Numerical	Total number of fallen trees observed
River/wetland depth	Average depth		Continuous	Centimeter (cm)
Water channel width	Water channel width			Meter (m)
Canopy cover (%)			Numerical	Percentage

^a^ Human Disturbance Index: 0–3 disturbance factors = low disturbance and above 3 disturbance factors = High disturbance.

Human disturbances like the presence of livestock grazing, solid waste, illegal sand mining, and fishing were used to generate two disturbance classes: high and low. This was measured and categorized based on the intensity of the disturbances (Table 1).

### 2.1 Data Analysis

The relationship between otter presence and habitat variables in the region was determined using a generalized linear model because of its flexibility to model continuous and categorical variables and non‐linear response (Grafen and Hails, 2002).

We used logistic regression for a binary outcome; logistic link and binomial distribution, to model the response of species to the selected environmental variables. We performed a multicollinearity test (Karl Pearson Correlation Coefficient) to remove information redundancy caused by the most related continuous variables (Table S1), and Cramer's V test to remove interdependent categorical values (Table S2) in R (Mangiafico 2025). We used a threshold of |0.7| to remove the least important variable among the pair above the threshold for both variable types (Table S1and S2). All the selected candidate environmental variables were then modeled using logistic regression with the “MuMIn” package (Barton and Barton 2015) in R version 4.1.3 (R Core Team 2023). We ran all the possible linear combinations of variables (Table S2) using the dredge function in the package. The top models were selected using the criteria of ΔAICc < 2 using the Akaike Information Criterion, which uses likelihood ratio penalized for the number of parameters to select the best model (Burnham and Anderson 2002). We checked the model assumptions and residual deviations from all the selected models using simulated data from the DHARMa package (Hartig 2024) in R before performing the model averaging. We used the significance level of 0.05 to infer the importance of a variable in the model‐averaged estimates.

## 3 Results

A total of 71 line transects were surveyed, with 53 conducted in the Chaudhar River and 18 in the wetlands. During the study period, a total of 33 transects with positive signs of otter presence were recorded, with 15 in the Chaudhar River and 18 in the wetlands (Table 2). Otter signs such as scats (11) and tracks (75) were recorded in rivers and wetlands inside the Park, while only one scat sign was found at the boundary of the protected area (the edge of the Chaudhar River).

**TABLE 2:** Presence of otter sign in the surveyed transect.

SN	Wetlands	Transect		Sign	
		Presence of sign	Absence of sign	Number	Percentage of sign presence
1	River	15	38	77	61
2	Lakes/marshy/wetlands	18	0	49	39
	Total	33	38	126	100

### 3.1 Physical Status of Wetlands and River

The average water depth in the wetland was 94.66 cm, while in the Chaudhar River, it measured 29.2 cm outside and 37.3 cm inside the Park. The average water channel width of the Chaudhar River was measured at 33.03 m. The water current in the wetland was stagnant with 75% of the location records indicating slow water flow. Similarly, 77% of locations in the Chaudhar River outside the protected area recorded slow water flow (Table 3). In terms of bank conditions, 83.3% of the wetlands observed no disturbance, compared to Chaudhar River locations both outside and inside the protected area. The majority of human disturbance (95.5%) was observed in the Chaudhar River located outside the protected area, compared to wetlands and rivers inside the protected area.

**TABLE 3:** Measurement of habitat variables in transects during field survey in wetlands and Chaudhar River inside and outside Shuklaphanta National Park (NP).

Variable	Measure	Wetlands	Chaudhar River outside NP	Chaudhar River inside NP
Average water depth (cm)	Numerical	94.66	29.23	37.39
Average width of water channel (m)	Numerical	442.16	33.03	30.89
Water current (%)	1. Slow	0	75	77.1
	2. Fast	0	25	22.9
	3. Stagnant	100		
Average bank slope (degree)	Numerical	40.31	35.71	43.91
Bank condition (%)	1. High disturbance	0	15	2.9
	2. Moderate disturbance	16.7	20	7.1
	3. No disturbance (good)	83.3	65	80
Bank substrate (%)	1. Sand and Mud (< 2 mm)	98.2	67.75	92.7
	2. Small stones (2‐64 mm)	1.8	20.75	4.86
	3. Big stones (64–256 mm)	0	11.5	2.7
	4. Boulder/rock (> 256 mm)	0	0	0
Human disturbance index (HDI %)	1. High	0	95.2	0
	2. Low	100	4.8	100
Human Disturbance factor (%)	1. Livestock grazing	0	25.4	5.6
	2. Illegal sand mining	0	15.5	0.0
	3. Fishing	0	23.9	2.8
	4. Solid waste(pollution)	0	19.7	11.3
	5. Infrastructure development	0	57.4	0
	6. Washing and bathing	0	66.7	0
	7. Human disturbances	0	25.4	4.2
Canopy cover (%)	0–25	16.7	81.0	25.0
	26–50	16.7	9.5	15.6
	Above 51	66.6	9.5	59.4
Shoreline vegetation (%)	Visual	98	52.4	96.9
Fallen tree (%)	Numerical	5.6	0	21.9

*Note:* % refers to the presence in the transect.

During the study period, canopy cover of more than 50% was observed as higher in the wetlands and Chaudhar River inside the protected area compared to sampling locations outside the protected area. Among the human disturbances, livestock grazing (25.4%) and illegal sand mining (15.5%) were notable. Fishing (23.9%), disposal of solid waste (19.7%), infrastructure development (57.4%), and washing and bathing (66.7%) were observed higher in the Chaudhar River outside the protected area compared to sampling areas inside ShNP (Table 3).

### 3.2 Factors Affecting Habitat Selection

A total of 128 models, incorporating single or multiple linear combinations of the selected variables, were utilized to ascertain the factors influencing the probability of smooth‐coated otter occurrence in the study area (Table S3). These variables included canopy cover (CC), human disturbance index, proportion of soil (PS) and mud in bank substrate (PS), width of water current (WCW), average depth of river, water current, and slope of bank (S). Among these models, only three showed some support for the presence of the species in the study area (Table 4; Figure S1–S3). The residuals did not deviate significantly from what was expected from the model (Figure S1, S2 and S3) .

**TABLE 4:** The most parsimonious (delAIC < 2) models used to determine the probability of the presence of smooth‐coated otter in Shuklaphanta National Park, Nepal.

Model	df	loglik	AICc	del AICc	Weight
CC + HDI + PS + WCW + S	7	−18.636	53.2	0	0.467
CC + HDI + WCW + S	6	−20.341	54.1	0.90	0.297
CC + PS + WCW + S	6	−20.573	54.6	1.37	0.236

Abbreviations: CC, canopy cover; HDI, Human Disturbance Index; PS, proportion of sand and mud in bank substrate; S, slope of bank; WCW, width of water current.

The cumulative model weight indicated that canopy cover, water channel width, and slope were the most important variables among the selected factors (cumulative model weight = 100%) showing a relationship with the occurrence of otters (Table 4). The Human Disturbance Index (HDI) (76.4%) and the proportion of sand and mud (PS) (70.3%) were identified as the next significant variables, contributing negatively and positively, respectively, to the presence of otters (Table 5). Water channel width emerged as a significant variable positively correlated with the presence of smooth‐coated otters (Table 5). Conversely, bank substrate, fallen trees, water depth, water current, and water with sandy/muddy beds played an insignificant role in the occurrence of otters.

Model‐averaged coefficient estimates highlighted that canopy cover, human disturbance index, and slope were the most significant factors influencing presence or absence. The likelihood of otters being present increased with higher canopy cover and in areas with low human disturbance and lower slopes, although the significance for HDI and slope was marginal (Table 5).

**TABLE 5:** Model‐averaged coefficients for the smooth‐coated otter presence in ShNP.

	Estimate	Adjusted SE	*z* value	Pr(>|*z*|)
(Intercept)	−6.49408	5.17171	1.256	0.20923
**CC2**	**2.11435**	**1.04479**	**2.024**	**0.043**
**CC3**	**4.32937**	**1.39675**	**3.1**	**0.00194**
**HDIL**	**1.88975**	**0.93164**	**2.028**	**0.04252**
PS	0.07998	0.05587	1.431	0.15232
WCW	0.02045	0.01249	1.637	0.10166
**S**	**−0.05446**	**0.02661**	**2.046**	**0.04071**

*Note:* Variables significantly contributing to the presence of the species are shown in bold text.

Abbreviations: CC, canopy cover; HDI, Human Disturbance Index; PS, proportion of sand and mud in bank substrate; S, slope of bank; WCW, width of water current.

## 4 Discussion

The study highlights the significant role of various environmental factors in determining the habitat suitability for smooth‐coated otters, particularly within Shuklaphanta National Park. Factors such as bank substrate composition (sand, mud, etc.), canopy cover, water channel width, riverbank slope, and human disturbance levels were identified as significant influencers of smooth‐coated otter presence. Among these, canopy cover emerged as the most important ecological variable in determining the distribution of the otters. Otters prefer substrates like sand and small stones for various activities. Our findings are consistent with previous studies, emphasizing the critical role of habitat variables and human disturbances in shaping otter distribution (Acharya et al. 2023; Kathariya et al. 2023; Gwachha et al. 2023; Basak et al. 2021). Our study observed a negative influence of increased riverbank slope on otter presence.

Shoreline vegetation plays a vital role in offering escape cover for otters during foraging and movement, while also providing important resting and denning sites (Hussain and Choudhury 1995; Anoop and Hussain 2004; Nawab and Hussain 2012a; Basak et al. 2021). Canopy‐covered banks with tall, mature trees provide sheltered riverbanks, stable temperatures, and diverse food resources (Khan et al. 2014; Chase et al. 2016; Virdana et al. 2024). Dense vegetation plays a crucial role in otter habitat selection, as noted in previous studies (Nawab 2007; Jayasurya et al. 2022; Preston et al. 2006; Thom et al. 1998; Hussain 2002; Nawab and Hussain 2012b). These areas also contribute nutrients and organic matter to watercourses, boosting food availability in aquatic ecosystems (Moun et al. 2024), which in turn support otter populations (Kausalya et al. 2006; Raha and Hussain 2016; Virdana et al. 2024).

Additionally, loose soil promotes otter presence, whereas hard soil negatively affects it (Shenoy et al. 2006). Shuklaphanta National Park, with its Chaudhar River, freshwater lakes like Rani Tal and Shikari Tal, and expansive grasslands and riparian vegetation, provides an essential habitat for smooth‐coated otters (Acharya et al. 2023). These findings offer important insights into the habitat preferences and factors that influence otter presence. The physical status of the wetlands and Chaudhar River demonstrated that areas with higher water depths and moderate to slower water currents inside ShNP provided more suitable habitats for otters. This aligns with Kruuk (2006), who highlighted that otters prefer areas with stable water levels and reduced human activities. Our study identified canopy cover, water channel width, and bank slope as the most significant variables influencing otter presence. This is consistent with the findings of Nawab and Hussain (2012a), who also reported the importance of these habitat characteristics in their study of otters in India, and Kathariya et al. (2023) and Acharya and Lamsal (2010) in Nepal. Gentle bank slopes are preferred, minimizing energy expenditure during foraging and grooming activities (Nawab and Hussain 2012a; Khan et al. 2014).

Precipitation in the driest month, warmest quarter, or driest quarter has a significant influence on habitat suitability for smooth‐coated otters in Nepal (Acharya et al. 2023). Grasslands and riverine forest habitats benefit from such precipitation, ensuring suitability for otters. However, decreased water levels affect wetlands and foraging grounds (Acharya 2017). Thus, minor changes in climate and land use variables could alter habitat suitability for otters (Acharya et al. 2023).

In ShNP and its wetlands, restricted fishing supports a stable otter population by mitigating anthropogenic pressures. High occurrences of otter indicators such as scats and tracks, especially within 10 m of the shoreline with sandy substrates, support previous findings (Khan et al. 2014; Joshi et al. 2021; Thapa et al. 2021). Awasthi et al. (2024) reported that otter presence in ShNP wetlands and nearby rivers is unaffected by microbial parameters. However, vegetation type and water quality significantly impact their presence. In the Terai's protected areas, riverine grasslands are dominated by *Saccharum spontaneum, Saccharum arundinaceum, Phragmites vallatoria, Erianthus ravennae, Imperata cylindrical*, and *Zizyphus rugosa*, providing crucial shelter for otters (Acharya et al. 2023). This underscores the importance of essential habitat characteristics for smooth‐coated otter conservation, emphasizing the necessity of large rivers and water bodies with managed prey bases and minimal human activities (Nawab and Hussain 2012a; Dias et al. 2022).

Smooth‐coated otter occurrence is primarily limited to protected areas within ShNP, rendering them vulnerable to activities beyond these boundaries. Illegal activities such as sand mining and unlicensed fishing, prohibited within Nepal's protected areas under the National Park and Wildlife Conservation Act of 1973, have heavily disturbed the river beyond the protected zone (Bashyal and Yadav 2020). Human disturbances such as livestock grazing, illegal sand mining, and infrastructure development, predominantly observed outside the protected area, negatively impact habitat suitability. This aligns with Weinberger et al. (2019), who emphasized the importance of restoring riparian vegetation to support otter populations in human‐dominated landscapes. Our study further highlights that the Human Disturbance Index is a critical negative factor affecting otter presence, as shown by the model‐averaged coefficients. No signs of otters were observed outside protected areas, particularly in the buffer zone and upstream of the Chaudhar River, due to anthropogenic disturbances (Acharya 2017; Gwachha et al. 2023). Fishing and sand/gravel extraction significantly contribute to their absence (Acharya and Lamsal 2010; Acharya et al. 2022). Shuklapantha, a wetland landscape, features a mix of habitats, with sparse canopy cover in open areas dominated by grasses and shrubs, contrasting with denser canopies in forested zones within the National Park. This variation supports diverse ecological functions: open wetlands enhance prey availability and thermoregulation for ShNP otters, while forested areas provide cover. However, the open structure increases otter visibility and vulnerability to human disturbance. The unique habitat dynamics of Shuklapantha highlight its ecological significance for otters, underscoring the need for tailored conservation strategies to mitigate human impacts and preserve this critical wetland ecosystem.

However, otters may inhabit areas without leaving spraints (scat) and can temporarily vacate a site but return later for marking or foraging (Hussain and Choudhury 1995; Acharya and Rajbhandari 2011; Nawab and Hussain 2012a). Informal interviews with local residents suggest that otters were once prevalent in the study area during the 1990s outside the protected zone. Habitat degradation due to human disturbance has led to a decline in habitat quality and subsequently, a decrease in the otter population. Despite these challenges, there is a high likelihood that otters could return with suitable habitat variables such as bank substrate composition, shoreline vegetation, and water body width. This is supported by findings in Koshi Tappu Wildlife Reserve, where photographic evidence showed otters reappearing after a decade (Mishra et al. 2022), and in the Rapti River of Chitwan National Park (pers. comm. Milan Tamang, 2023). Deforestation, changes in canopy cover, and the conversion of riparian vegetation for agriculture affect the suitability of habitat for smooth‐coated otters (Acharya et al. 2023) Restoring riparian vegetation is essential for supporting otters in human‐dominated landscapes (Weinberger et al. 2019; Acharya et al. 2023).

The study underscores the need for active conservation measures, including research, habitat protection, and aquatic species management, to ensure the survival of smooth‐coated otters. Human disturbances like illegal fishing, sand, boulder collection, and grazing were prevalent near the Chaudhar River human settlement site. Urgent conservation needs include field research, monitoring, stringent habitat protection, and aquatic species management protocols.

## 5 Conclusion

Smooth‐coated otters were predominantly observed within the protected areas of Shuklaphanta National Park. Their presence was positively influenced by canopy cover, a higher proportion of sand and mud, and wider river channels. In contrast, otter occurrence declined with increased human disturbance and steeper riverbank slopes, while water depth and current had no significant effect. Outside the Park, suitable habitats are heavily impacted by anthropogenic disturbances, limiting otter distribution. Conservation efforts should focus on mitigating human activities, enhancing habitat quality, and raising community awareness. Further research on habitat preferences and regular monitoring of water quality and vegetation are essential to support otter conservation both within and beyond protected areas. Regular monitoring of water quality and vegetation, along with efforts to reduce anthropogenic activities, is urgently needed to conserve the smooth‐coated otter and its habitat in the western lowlands of Nepal.

## Author Contributions

**Balram Awasthi:** conceptualization (lead), data curation (lead), formal analysis (supporting), funding acquisition (lead), investigation (lead), methodology (lead), project administration (lead), resources (lead), visualization (lead), writing – original draft (lead), writing – review and editing (equal). **Suraj Baral:** formal analysis (lead), writing – original draft (supporting), writing – review and editing (equal). **Grace M. Yoxon:** writing – review and editing (equal). **Babu Ram Banjade:** methodology (supporting), writing – review and editing (equal). **Purna Man Shrestha:** conceptualization (equal), data curation (equal), writing – original draft (equal), writing – review and editing (equal).

## Conflicts of Interest

The authors declare no conflicts of interest.

## Supporting information


Data S1.

## Data Availability

The dataset includes detailed information on habitat selection, environmental parameters, and analysis scripts used in the study are openly available in Dryad under the DOI: https://doi.org/10.5061/dryad.2v6wwq00g.

## References

[ece371297-bib-0001] Acharya, P. M. 1998. “Survey of Status and Distribution of Otter in Rapti River of Royal Chitwan National Park.” A Report Submitted to Otter Research Group, Japan.

[ece371297-bib-0002] Acharya, P. M. 2006. “Otter and Wetland Conservation in Nepal.” In Water Resources, Security and Sustainability, 144–149. SEEP Water.

[ece371297-bib-0003] Acharya, P. M. 2017. “Status of Smooth‐Coated Otters *Lutrogale perspicillata* (Geoffroy, 1826) in the Khauraha River of Bardia National Park, Nepal.” OTTER, Journal of the International Otter Survival Fund 3: 23–37.

[ece371297-bib-0004] Acharya, P. M. , and P. Lamsal . 2010. “A Survey for Smooth‐Coated Otter (*Lutrogale perspicillata*) on the River Narayani, Chitwan National Park, Nepal.” Hystrix Italian Journal of Mammalogy 21, no. 2: 199–202. 10.4404/Hystrix-21.2-4464.

[ece371297-bib-0005] Acharya, P. M. , and S. Rajbhandari . 2011. “Distribution and Conservation Status of Otters in Nepal.” Zoo Journal 2: 27–37.

[ece371297-bib-0006] Acharya, P. M. , and S. L. Rajbhandari . 2012. “Status and Conservation of Otters in Nepal.” Hamro Kalpabrichha 33, no. 256: 16–21.

[ece371297-bib-0007] Acharya, P. M. , and S. L. Rajbhandari . 2014. “Habitats of *Lutrogale perspicillata* in the Narayani River, Chitwan National Park, Nepal: Assessment of Water Quality.” Journal of Indian Research 2: 67–76.

[ece371297-bib-0008] Acharya, P. M. , S. Saeung , K. Techato , N. Rimal , S. Gyawali , and D. Neupane . 2022. “Review of Environmental Policies and Otter Conservation in Nepal.” IUCN Otter Specialist Group Bulletin 39, no. 1: 44–55.

[ece371297-bib-0009] Acharya, P. M. , P. Thainiramit , K. Techato , et al. 2023. “Predicting the Distribution and Habitat Suitability of the Smooth‐Coated Otter ( *Lutrogale perspicillata* ) in Lowland Nepal.” Global Ecology and Conservation 46: e02578. 10.1016/j.gecco.2023.e02578.

[ece371297-bib-0101] Basnet, A. , B. S. Bist , P. Ghimire , and P. M. Acharya . 2020. “Eurasian Otter (*Lutra lutra*): Exploring Evidence in Nepal.” IUCN Otter Specialist Group Bulletin 37, no. 1: 29–37.

[ece371297-bib-0010] Burnham, K. P. , and D. R. Anderson . 2002. Model Selection and Multimodel Inference: A Practical Information‐Theoretic Approach. 2nd ed. Springer‐Verlag.

[ece371297-bib-0011] Anoop, K. , and S. Hussain . 2004. “Factors Affecting Habitat Selection by Smooth‐Coated Otters (*Lutra perspicillata*) in Kerala, India.” Journal of Zoology 263, no. 4: 417–423. 10.1017/S095283964005461.

[ece371297-bib-0012] Arlt, D. 2007. “Habitat Selection: Demography and Individual Decisions.” Dissertation. Department of Ecology, Swedish University of Agricultural Sciences.

[ece371297-bib-0013] Awasthi, B. , B. Banjade , N. Pandey , et al. 2024. “The Effects of Biological Water Quality on the Presence of the Smooth‐Coated Otter in Far Western Nepal.” IUCN Otter Specialist Group Bulletin 41, no. 2: 40–56.

[ece371297-bib-0014] Barton, K. , and M. K. Barton . 2015. “Package ‘MuMIn’. Version.” 1 (18), 439.

[ece371297-bib-0015] Basak, S. , B. Pandav , A. J. Johnson , and S. A. Hussain . 2021. “Resource Utilisation by Smooth‐Coated Otters in the Rivers of Himalayan Foothills in Uttarakhand, India.” Global Ecology and Conservation 32: e01896. 10.1016/j.gecco.2021.e01896.

[ece371297-bib-0016] Bashyal, A. , and B. Yadav . 2020. “Opportunistic Smooth‐Coated Otter (*Lutrogale perspicillata*) Sightings Record in the Bardiya National Park of Nepal.” IUCN Otter Specialist Group Bulletin 37, no. 2: 120–126.

[ece371297-bib-0018] Bhandari, J. 2019. “Conservation Status Survey and Awareness of Smooth‐Coated Otters in Babai River of Bardia National Park, Nepal.” A Final Report Submitted to Rufford Small Grants Foundation, UK.

[ece371297-bib-0020] Chase, J. W. , G. A. Benoy , S. W. R. Hann , and J. M. Culp . 2016. “Small Differences in Riparian Vegetation Significantly Reduce Land Use Impacts on Stream Flow and Water Quality in Small Agricultural Watersheds.” Journal of Soil and Water Conservation 71, no. 3: 194–205. 10.2489/JSWC.71.3.194.

[ece371297-bib-0021] CITES . 2021. “Conventional International Trade in Endangered Species of Wild Fauna and Flora.” https://cites.org/sites/default/files/eng/app/2021/E‐Appendices‐2021‐06‐22.pdf.

[ece371297-bib-0022] de Silva, P. , W. A. Khan , B. Kanchanasaka , I. Reza Lubis , M. M. Feeroz , and O. F. Al‐Sheikhly . 2015. “ *Lutrogale perspicillata* .” In The IUCN Red List of Threatened Species 2015. International Union for Conservation of Nature (IUCN). https://www.iucnredlist.org.

[ece371297-bib-0023] Department of National Park and Wildlife Conservation . 2003. Royal Shuklaphanta Wildlife Reserve Management Plan. Department of National Parks and Conservation.

[ece371297-bib-0024] Dias, S. , J. C. White , A. S. Borker , and N. V. Fernandez . 2022. “Habitat Selection of Smooth‐Coated Otters (*Lutrogale perspicillata*) in the Peri‐Coastal, Urbanized Landscape of Goa, India.” Mammal Research 67, no. 3: 299–309. 10.1007/s13364-022-00639-1.

[ece371297-bib-0027] Government of Nepal . 2017. “Aquatic Animal Protection Act, 2017 (1960).” http://www.lawcommission.gov.np.

[ece371297-bib-0025] Grafen, A. , and R. Hails . 2002. Modern statistics for the life sciences. Oxford University Press.

[ece371297-bib-0028] Gwachha, S. , M. Koirala , and P. M. Shrestha . 2023. “Habitat Status of the Smooth‐Coated Otter (*Lutrogale perspicillata*) in Geruwa‐Khaurahi River, Bardia National Park, Nepal.” Nepal Journal of Environmental Science 11, no. 2: 23–33. 10.3126/njes.v11i2.56115.

[ece371297-bib-0029] Hartig, F. 2024. “DHARMa: Residual Diagnostics for Hierarchical (Multi‐Level/Mixed) Regression Models. R Package Version 0.4.7.” https://CRAN.R‐project.org/package=DHARMa.

[ece371297-bib-0030] Hodgson, B. H. 1839. “Summary Description of Four New Species of Otter.” Journal of the Asiatic Society of Bengal 8: 319–320.

[ece371297-bib-0031] Hussain, S. A. 1996. “Group Size, Group Structure and Breeding in Smooth‐Coated Otter (*Lutra perspicillata* Geoffroy) in National Chambal Sanctuary.” Mammalia 60: 289–297.

[ece371297-bib-0032] Hussain, S. A. 2002. “Conservation Status of Otters in the Tarai and Lower Himalayas of Uttar Pradesh, India.” In Otter Conservation—An Example for a Sustainable Use of Etlands, edited by R. Dulfer , J. Conroy , J. Nel , and A. Gutleb , 131–142. IUCN Otter Specialist Group Bulletin 19 Special Issue A Part II.

[ece371297-bib-0033] Hussain, S. A. , and B. C. Choudhury . 1995. “Seasonal Movement, Home Range, and Habitat Use by Smooth‐Coated Otters in National Chambal Sanctuary, India.” In Habitat 11, Proceedings of the VIth International Otter Colloquium, Pietermaritzburg 1993, edited by C. Reuther and D. Rowe‐Rowe , 45–55. Aktion Fischotterschutz.

[ece371297-bib-0034] IUCN . 2024. “The IUCN Red List of Threatened Species (Version 3.1).” https://www.iucnredlist.org.

[ece371297-bib-0035] Jamwal, P. S. , J. Takpa , P. Chandan , and M. Savage . 2016. “First Systematic Survey for Otter (*Lutra lutra*) in Ladakh, Indian Trans Himalayas.” IUCN Otter Specialist Group Bulletin 33, no. 2: 79–85.

[ece371297-bib-0036] Jayasurya, M. , M. Moorthi , S. Sathishkumar , and R. Srimathi . 2022. “Distribution, Habitat Selection and Diet of Mooth‐Coated Otters (*Lutrogale perspicillata*) in the Kollidam and Thenpennai Rivers in South India.” IUCN Otter Specialist Group Bulletin 40, no. D: 3–15.

[ece371297-bib-0037] Jnawali, S. R. , H. S. Baral , S. Lee , et al. 2011. The Status of Nepal Mammals: The National Red List Series. Department of National Parks and Wildlife Conservation Kathmandu.

[ece371297-bib-0074] Joshi, G. K. , R. Joshi , and B. Poudel . 2021. “Distribution and Threats to Smooth‐Coated Otters *Lutrogale perspicillata* (Mammalia: Carnivora: Mustelidae) in Shuklaphanta National Park, Nepal.” Journal of Threatened Taxa 13, no. 11: 19475–19483. 10.11609/jott.7322.13.11.19475-19483.

[ece371297-bib-0038] Kathariya, R. , D. R. Pant , K. R. Gosai , R. P. Sapkota , and M. B. Shrestha . 2023. “Effects of Habitat Variables on the Distribution of Smooth‐Coated Otters (*Lutrogale perspicillata*) Along the Kauriala Branch of the Karnali River, Nepal.” IUCN Otter Specialist Group Bulletin 41, no. 2: 40–56.

[ece371297-bib-0039] Kausalya, S. , V. Surendra , and K. V. Devi Prasad . 2006. “Factors Determining Habitat Choice of the Smooth‐Coated Otter, *Lutra perspicillata* in a SouthIndian River System.” Current Science 91, no. 5: 637–643.

[ece371297-bib-0040] Khan, M. S. , N. K. Dimri , K. A. Nawab , O. Ilyas , and P. Gautam . 2014. “Habitat Use Pattern and Conservation Status of Smooth–Coated Otters *Lutrogale perspicillata* in the Upper Ganges Basin, India.” Animal Biodiversity and Conservation 37, no. 1: 69–76.

[ece371297-bib-0041] Khoo, M. , S. Basak , N. Sivasothi , P. K. de Silva , and I. Reza Lubis . 2021. “ *Lutrogale perspicillata* .” In The IUCN Red List of Threatened Species 2021. e. T12427A164579961. International Union for Conservation of Nature (IUCN). 10.2305/IUCN.UK.2021-3.RLTS.T12427A164579961.e.

[ece371297-bib-0042] Kruuk, H. 2006. Otters: Ecology, Behavior and,Conservation. Oxford University Press.

[ece371297-bib-0043] Kruuk, H. , and J. W. H. Conroy . 1987. “Surveying Otter *Lutra lutra* Populations: A Discussion of Problems With Spraints.” Biological Conservation 41, no. 3: 179–183. 10.1016/0006-3207(87)90101-7.

[ece371297-bib-0044] Madsen, A. B. , and A. Prang . 2001. “Habitat Factors and the Presence or Absence of Otters *Lutra lutra* in Denmark.” Acta Theriologica 46, no. 2: 171–179. 10.1007/BF03192426.

[ece371297-bib-0076] Mangiafico, S. S. 2025. “rcompanion: Functions to Support Extension Education Program Evaluation.” Rutgers Cooperative Extension, New Brunswick, NJ. Version 2.5.0. https://CRAN.R‐project.org/package=rcompanion/.

[ece371297-bib-0045] Mason, C. F. , and S. M. Macdonald . 1986. “The Use of Spraints for Surveying Otter *Lutra lutra* Population: An Evaluation.” Biological Conservation 41, no. 1987: 167–177.

[ece371297-bib-0046] Melisch, R. , L. Kusumawardhani , P. B. Asmoro , and I. R. Lubis . 1996. The Otters of West Java—A Survey of Their Distribution and Habitat Use and a Strategy Towards a Species Conservation Programme. PHPA/Wetlands International – Indonesia Programme.

[ece371297-bib-0048] Mishra, R. , B. R. Lamichhane , B. Gautum , A. K. Ram , and N. Subedi . 2022. “Photographic Evidence of Smooth‐Coated Otter *Lutrogale perspicillata* in Koshi Tappu Wildlife Reserve, Nepal.” IUCN Otter Specialist Group Bulletin 39, no. 4: 189–195.

[ece371297-bib-0049] Morrison, M. L. , B. G. Marcot , and R. W. Mannan . 1992. Wildlife–Habitat Relationships: Concept and Applications. University of Wisconsin Press.

[ece371297-bib-0050] Moser, M. , C. Prentice , and S. Frazier . 1996. “A Global Overview of Wetland Loss and Degradation.” Proceedings of the 6th Meeting of the Conference of the Contracting Parties to the Ramsar Convention on Wetlands, 19–27 March 1996, Brisbane, Australia.

[ece371297-bib-0051] Moun, A. , P. R. Kumar , M. M. Priya , T. Ramesh , and R. Kalle . 2024. “Multi‐Scale Habitat Influences Sprainting and Group Size of a Freshwater‐Obligate Smooth‐Coated Otter (*Lutrogale perspicillata*) in Tungabhadra Otter Conservation Reserve, India.” Ecological Processes 13, no. 1: 1–17. 10.1186/s13717-024-00492-x.

[ece371297-bib-0052] Nawab, A. 2007. “Ecology of Otters in Corbett Tiger Reserve, Uttarakhand; India.” PhD Thesis, Forest Research Institute.

[ece371297-bib-0053] Nawab, A. , and S. A. Hussain . 2012a. “Factors Affecting the Occurrence of Smooth‐Coated Otter in Aquatic Systems of the Upper Gangetic Plains, India.” Aquatic Conservation: Marine and Freshwater Ecosystems 22, no. 5: 616–625.

[ece371297-bib-0054] Nawab, A. , and S. A. Hussain . 2012b. “Prey Selection by Smooth‐Coated Otter (*Lutrogale perspicillata*) in Response to the Variation in Fish Abundance in Upper Gangetic Plains, India.” Mammalia 76, no. 1: 57–65. 10.1515/mamm.2011.105.

[ece371297-bib-0055] Poudyal, L. P. , and H. Chaudhary . 2019. Birds of Shuklaphanta National Park. Shuklaphanta National Park Office and Nepalese Ornithological Union.

[ece371297-bib-0056] Poudyal, L. P. , Y. B. Rawat , D. K. Yadav , D. R. Joshi , and K. R. Bohara . 2021. “First Report of Himalayan Goral From Nepal's Shuklaphanta National Park.” Mammal Tales 24: 23–25.

[ece371297-bib-0057] Preston, S. J. , A. A. Portig , W. I. Montgomery , R. A. McDonald , and J. S. Fairley . 2006. “Status and Diet of the Otter *Lutra lutra* in Northern Ireland.” Biology and Environment: Proceedings of the Royal Irish Academy 106, no. 1: 57–63. 10.3318/BIOE.2006.106.1.56.

[ece371297-bib-0058] R Core Team . 2023. R: A Language and Environment for Statistical Computing. R Foundation for Statistical Computing. https://www.R‐project.org/.

[ece371297-bib-0059] Raha, A. , and S. A. Hussain . 2016. “Factors Affecting Habitat Selection by Three Sympatric Otter Species in the Southern Western Ghats, India.” Acta Ecologica Sinica 36: 45–49. 10.1016/j.chnaes.2015.12.002.

[ece371297-bib-0060] Rawat, Y. B. , S. Bhattarai , L. P. Poudyal , and N. Subedi . 2020. “Herpetofauna of Shuklaphanta National Park, Nepal.” Journal of Threatened Taxa 12, no. 5: 15587–15611. 10.11609/jott.

[ece371297-bib-0061] Russavage, E. , J. Thiele , J. Lumbsden‐Pinto , K. Schwager , T. Green , and M. Dovciak . 2021. “Characterizing Canopy Openness in Open Forests: Spherical Densiometer and Canopy Photography Are Equivalent but Less Sensitive Than Direct Measurements of Solar Radiation.” Journal of Forestry 119, no. 2: 130–140.

[ece371297-bib-0062] Savage, M. , and M. Shrestha . 2018. “The Illegal Trade in Otter Pelts in Nepal.” TRAFFIC Bulletin 30, no. 2: 59–63.

[ece371297-bib-0063] Shenoy, K. , S. Varma , and K. D. Prasad . 2006. “Factors Determining Habitat Choice of the Smooth‐Coated Otter (*Lutragale perspicillata*) in a South Indian River System.” Current Science 91: 637–643. http://eprints.iisc.ac.in/8831/1/Factors_determining_habitat_choice_of_the.pdf.

[ece371297-bib-0064] Shrestha, M. B. , G. Shrestha , S. Reule , S. Oli , D. M. Tripathi , and M. Savage . 2021. “Otter Survey Along the Sanibheri River and Its Tributaries, the Pelma and Utterganga Rivers in Rukum District, Western Nepal.” IUCN Otter Specialist Group Bulletin 38, no. 5: 267–278.

[ece371297-bib-0065] Shrestha, M. B. , G. Shrestha , H. L. Dangaura , et al. 2025. “Confirmation of the Presence of Asian Small‐Clawed Otter (*Aonyx cinereus*) in Nepal After 185 Years.” IUCN Otter Specialist Group Bulletin 42, no. 1: 3–8.

[ece371297-bib-0066] Thapa, P. , G. C. D. Bijaya , J. Bhandari , B. P. Devkota , T. Silwal , and L. Can . 2021. “Distribution, Threats and Community Perceptions of Otters in Shuklaphanta National Park, Nepal.” OTTER, Journal of the International Otter Survival Fund 7: 128–142.

[ece371297-bib-0067] Thom, T. J. , C. J. Thomas , N. Dunstone , and P. R. Evans . 1998. “The Relationship Between Riverbank Habitat and Prey Availability and the Distribution of Otter ( *Lutra lutra* ) Signs: An Analysis Using a Geographical Information System.” In Behaviour and Ecology of Riparian Mammals, 135–158. Cambridge University Press. ISBN 9780511721830. 10.1017/CBO9780511721830.010.

[ece371297-bib-0068] Virdana, S. , F. Andeska , A. Aadrean , et al. 2024. “Habitat Use Overlap Among Three Otter Species (*Lutra lutra*, *Lutra sumatrana*, and *Aonyx cinereus*) in the Dharmasraya Sumatran Tiger Rehabilitation Centre Area, West Sumatra, Indonesia.” IUCN Otter Specialist Group Bulletin 41, no. 2: 64–70.

[ece371297-bib-0069] Weinberger, I. C. , S. Muff , A. Kranz , and F. Bontadina . 2019. “Riparian Vegetation Provides Crucial Shelter for Resting Otters in a Human‐Dominated Landscape.” Mammalian Biology 98: 179–187. 10.1016/j.mambio.2019.09.001.

[ece371297-bib-0071] Yoxon, G. , and P. Yoxon . 1990. “Otter Survey of the Isle of Skye, Scotland (*Lutra lutra*).” IUCN Otter Specialist Group Bulletin 5: 70–75.

