# Supplementary material for: Habitat Selection of the Smooth‐Coated Otter ( Lutrogale perspicillata ) in Shuklaphanta National Park, Nepal
Source: Ecol Evol. 2025 May 26;15(5):e71297. doi: 10.1002/ece3.71297 (PMC12104665; doi:10.1002/ece3.71297)
Supplement: Supplementary file 1 — Data S1. [file ECE3-15-e71297-s001.docx]

# **Habitat Selection of the Smooth-coated Otter (*Lutrogale perspicillata*) in Shuklaphanta National Park, Nepal**

Balram AWASTHI^1^ Suraj BARAL^2,3^, Babu Ram BANJADE ^4^, Grace M YOXON ^5^, Purna Man SHRESTHA ^4,6,7*^

^1^ Department of Zoology, Siddhanath Science Campus, Tribhuvan University, Mahendranagar, Nepal

^2^ Biodiversity Research and Conservation Society, Kathmandu, Nepal

^3^ LIB, Museum Koenig, Leibniz Institute for the Analysis of Biodiversity Change, Adenauerallee 127, 53113 Bonn, Germany

^4^ Nepal Zoological Society, Kirtipur, Kathmandu, Nepal

^5^ International Otter Survival Fund, 7 Black Park, Broadford, Isle of Skye IV49 9DE, United Kingdom

^6^ Wildlife Research and Education Network, Kathmandu, Nepal

^7^IUCN Species Survival Commission, Otter Specialist Group, Rue Mauverney 28, 1196 Gland, Switzerland

**Supplemental material**

**Table S1. Correlation coefficients among continuous predictors used in modeling habitat selection of the Smooth-coated otter (*Lutrogale perspicillata*) in Shuklaphanta National Park, Nepal**

|  | slope | prop_soil | prop_pebble | prop_stone | avg_dpt | mid_dpt | river_channel |
| --- | --- | --- | --- | --- | --- | --- | --- |
| slope | 1.000 |  |  |  |  |  |  |
| prop_soil | 0.206 | 1.000 |  |  |  |  |  |
| prop_pebble | -0.064 | -0.849 | 1.000 |  |  |  |  |
| prop_stone | -0.285 | -0.855 | 0.453 | 1.000 |  |  |  |
| avg_dpt | -0.039 | 0.034 | 0.157 | -0.211 | 1.000 |  |  |
| mid_dpt | -0.013 | 0.034 | 0.133 | -0.189 | 0.947 | 1.000 |  |
| river_channel | 0.016 | 0.152 | -0.128 | -0.131 | 0.344 | 0.413 | 1.000 |

**Table S2. Correlation coefficients among categorical predictors used in modeling habitat selection of the Smooth-coated otter (*Lutrogale perspicillata*) in Shuklaphanta National Park, Nepal**

| Kramers' V correlation coefficient for categorical variables | | | | |
| --- | --- | --- | --- | --- |
|  | habitat | cc | current | HDI |
| habitat | 1 |  |  |  |
| cc | 0.396 | 1 |  |  |
| current | 0.763 | 0.298 | 1 |  |
| HDI | 0.783 | 0.41 | 0.582 | 1 |

**Table S3. Summary of all models evaluated using the dredge function for Habitat Selection of the Smooth-Coated Otter (Lutrogale perspicillata) in Shuklaphanta National Park, Nepal**

| . | (Intercept) | avg_dpt | cc | current | HDI | prop_soil | river_channel | slope | df | logLik | AICc | delta | weight |
| --- | --- | --- | --- | --- | --- | --- | --- | --- | --- | --- | --- | --- | --- |
| 123 | -7.608 | NA | + | NA | + | 0.070 | 0.019 | -0.058 | 7 | -18.636 | 53.203 | 0.000 | 0.194 |
| 107 | -2.072 | NA | + | NA | + | NA | 0.023 | -0.052 | 6 | -20.341 | 54.105 | 0.902 | 0.124 |
| 115 | -9.865 | NA | + | NA | NA | 0.099 | 0.020 | -0.051 | 6 | -20.573 | 54.570 | 1.367 | 0.098 |
| 124 | -7.546 | -0.003 | + | NA | + | 0.071 | 0.020 | -0.059 | 8 | -18.618 | 55.763 | 2.560 | 0.054 |
| 127 | -7.247 | NA | + | + | + | 0.072 | 0.022 | -0.068 | 9 | -17.556 | 56.326 | 3.123 | 0.041 |
| 59 | -9.187 | NA | + | NA | + | 0.067 | 0.015 | NA | 6 |  | 56.541 | 3.338 | 0.037 |
| 108 | -2.043 | -0.001 | + | NA | + | NA | 0.023 | -0.053 | 7 | -20.339 | 56.609 | 3.406 | 0.035 |
| 111 | -1.429 | NA | + | + | + | NA | 0.026 | -0.064 | 8 | -19.151 | 56.828 | 3.625 | 0.032 |
| 43 | -3.516 | NA | + | NA | + | NA | 0.018 | NA | 5 | -22.933 | 56.865 | 3.662 | 0.031 |
| 51 | -10.365 | NA | + | NA | NA | 0.087 | 0.016 | NA | 5 | -22.953 | 56.906 | 3.703 | 0.030 |
| 116 | -9.869 | 0.000 | + | NA | NA | 0.099 | 0.020 | -0.050 | 7 | -20.573 | 57.076 | 3.873 | 0.028 |
| 91 | -8.290 | NA | + | NA | + | 0.089 | NA | -0.050 | 6 | -22.032 | 57.487 | 4.284 | 0.023 |
| 95 | -9.047 | NA | + | + | + | 0.096 | NA | -0.048 | 8 | -19.529 | 57.584 | 4.381 | 0.022 |
| 92 | -8.852 | 0.015 | + | NA | + | 0.085 | NA | -0.047 | 7 | -21.079 | 58.088 | 4.885 | 0.017 |
| 99 | -1.589 | NA | + | NA | NA | NA | 0.023 | -0.044 | 5 | -23.591 | 58.181 | 4.978 | 0.016 |
| 128 | -7.389 | -0.011 | + | + | + | 0.079 | 0.023 | -0.070 | 10 | -17.372 | 58.744 | 5.541 | 0.012 |
| 60 | -9.260 | 0.006 | + | NA | + | 0.066 | 0.014 | NA | 7 | -21.463 | 58.858 | 5.655 | 0.011 |
| 119 | -10.328 | NA | + | + | NA | 0.105 | 0.018 | -0.047 | 8 | -20.171 | 58.869 | 5.666 | 0.011 |
| 52 | -10.523 | 0.008 | + | NA | NA | 0.086 | 0.015 | NA | 6 | -22.745 | 58.913 | 5.710 | 0.011 |
| 31 | -10.432 | NA | + | + | + | 0.090 | NA | NA | 7 | -21.600 | 59.131 | 5.928 | 0.010 |
| 44 | -3.623 | 0.003 | + | NA | + | NA | 0.017 | NA | 6 | -22.890 | 59.204 | 6.001 | 0.010 |
| 112 | -1.072 | -0.008 | + | + | + | NA | 0.027 | -0.065 | 9 | -19.001 | 59.217 | 6.014 | 0.010 |
| 35 | -2.867 | NA | + | NA | NA | NA | 0.019 | NA | 4 | -25.468 | 59.593 | 6.390 | 0.008 |
| 23 | -12.279 | NA | + | + | NA | 0.115 | NA | NA | 6 | -23.129 | 59.682 | 6.479 | 0.008 |
| 63 | -9.355 | NA | + | + | + | 0.072 | 0.013 | NA | 8 | -20.612 | 59.751 | 6.548 | 0.007 |
| 55 | -10.986 | NA | + | + | NA | 0.095 | 0.013 | NA | 7 | -21.949 | 59.828 | 6.625 | 0.007 |
| 28 | -9.839 | 0.018 | + | NA | + | 0.076 | NA | NA | 6 | -23.232 | 59.887 | 6.684 | 0.007 |
| 47 | -3.268 | NA | + | + | + | NA | 0.016 | NA | 7 | -22.064 | 60.059 | 6.856 | 0.006 |
| 79 | -1.033 | NA | + | + | + | NA | NA | -0.040 | 7 | -22.088 | 60.108 | 6.905 | 0.006 |
| 87 | -12.106 | NA | + | + | NA | 0.128 | NA | -0.031 | 7 | -22.111 | 60.152 | 6.949 | 0.006 |
| 96 | -9.217 | -0.003 | + | + | + | 0.099 | NA | -0.048 | 9 | -19.510 | 60.234 | 7.031 | 0.006 |
| 27 | -9.390 | NA | + | NA | + | 0.080 | NA | NA | 5 | -24.643 | 60.285 | 7.082 | 0.006 |
| 100 | -1.565 | -0.001 | + | NA | NA | NA | 0.023 | -0.044 | 6 | -23.590 | 60.603 | 7.400 | 0.005 |
| 15 | -2.543 | NA | + | + | + | NA | NA | NA | 6 | -23.674 | 60.772 | 7.569 | 0.004 |
| 84 | -11.387 | 0.020 | + | NA | NA | 0.115 | NA | -0.033 | 6 | -23.784 | 60.992 | 7.789 | 0.004 |
| 76 | -2.024 | 0.016 | + | NA | + | NA | NA | -0.038 | 6 | -23.811 | 61.045 | 7.842 | 0.004 |
| 20 | -11.594 | 0.021 | + | NA | NA | 0.103 | NA | NA | 5 | -25.071 | 61.141 | 7.938 | 0.004 |
| 120 | -10.664 | -0.011 | + | + | NA | 0.113 | 0.019 | -0.048 | 9 | -19.988 | 61.190 | 7.987 | 0.004 |
| 75 | -1.215 | NA | + | NA | + | NA | NA | -0.037 | 5 | -25.177 | 61.353 | 8.150 | 0.003 |
| 121 | -9.987 | NA | NA | NA | + | 0.112 | 0.009 | -0.047 | 5 | -25.192 | 61.385 | 8.182 | 0.003 |
| 32 | -10.826 | -0.007 | + | + | + | 0.096 | NA | NA | 8 | -21.536 | 61.598 | 8.396 | 0.003 |
| 89 | -10.490 | NA | NA | NA | + | 0.121 | NA | -0.048 | 4 | -26.568 | 61.792 | 8.589 | 0.003 |
| 36 | -2.998 | 0.004 | + | NA | NA | NA | 0.018 | NA | 5 | -25.407 | 61.814 | 8.611 | 0.003 |
| 12 | -3.277 | 0.016 | + | NA | + | NA | NA | NA | 5 | -25.461 | 61.922 | 8.719 | 0.002 |
| 24 | -12.635 | -0.006 | + | + | NA | 0.121 | NA | NA | 7 | -23.073 | 62.077 | 8.874 | 0.002 |
| 56 | -11.300 | -0.009 | + | + | NA | 0.101 | 0.013 | NA | 8 | -21.811 | 62.149 | 8.946 | 0.002 |
| 64 | -9.670 | -0.010 | + | + | + | 0.078 | 0.014 | NA | 9 | -20.479 | 62.172 | 8.969 | 0.002 |
| 48 | -2.977 | -0.008 | + | + | + | NA | 0.017 | NA | 8 | -21.920 | 62.366 | 9.163 | 0.002 |
| 103 | -1.657 | NA | + | + | NA | NA | 0.020 | -0.038 | 7 | -23.224 | 62.379 | 9.176 | 0.002 |
| 11 | -2.502 | NA | + | NA | + | NA | NA | NA | 4 | -26.878 | 62.412 | 9.209 | 0.002 |
| 83 | -10.832 | NA | + | NA | NA | 0.122 | NA | -0.037 | 5 | -25.725 | 62.450 | 9.247 | 0.002 |
| 39 | -2.833 | NA | + | + | NA | NA | 0.015 | NA | 6 | -24.517 | 62.458 | 9.255 | 0.002 |
| 80 | -1.075 | 0.001 | + | + | + | NA | NA | -0.040 | 8 | -22.085 | 62.696 | 9.493 | 0.002 |
| 88 | -12.324 | -0.003 | + | + | NA | 0.131 | NA | -0.031 | 8 | -22.090 | 62.705 | 9.503 | 0.002 |
| 93 | -10.759 | NA | NA | + | + | 0.120 | NA | -0.040 | 6 | -24.820 | 63.064 | 9.861 | 0.001 |
| 16 | -2.458 | -0.002 | + | + | + | NA | NA | NA | 7 | -23.663 | 63.257 | 10.054 | 0.001 |
| 90 | -10.740 | 0.009 | NA | NA | + | 0.119 | NA | -0.045 | 5 | -26.146 | 63.292 | 10.089 | 0.001 |
| 19 | -11.095 | NA | + | NA | NA | 0.109 | NA | NA | 4 | -27.365 | 63.386 | 10.183 | 0.001 |
| 122 | -10.039 | 0.002 | NA | NA | + | 0.112 | 0.008 | -0.046 | 6 | -25.173 | 63.770 | 10.567 | 0.001 |
| 57 | -12.494 | NA | NA | NA | + | 0.122 | 0.008 | NA | 4 | -27.602 | 63.859 | 10.656 | 0.001 |
| 29 | -12.730 | NA | NA | + | + | 0.126 | NA | NA | 5 | -26.488 | 63.976 | 10.773 | 0.001 |
| 7 | -2.058 | NA | + | + | NA | NA | NA | NA | 5 | -26.687 | 64.374 | 11.171 | 0.001 |
| 104 | -1.258 | -0.009 | + | + | NA | NA | 0.021 | -0.039 | 8 | -23.010 | 64.547 | 11.344 | 0.001 |
| 40 | -2.490 | -0.009 | + | + | NA | NA | 0.016 | NA | 7 | -24.318 | 64.566 | 11.363 | 0.001 |
| 25 | -12.771 | NA | NA | NA | + | 0.128 | NA | NA | 3 | -29.112 | 64.611 | 11.408 | 0.001 |
| 125 | -10.274 | NA | NA | + | + | 0.113 | 0.007 | -0.042 | 7 | -24.441 | 64.813 | 11.610 | 0.001 |
| 94 | -10.968 | -0.009 | NA | + | + | 0.125 | NA | -0.040 | 7 | -24.647 | 65.225 | 12.022 | 0.000 |
| 21 | -14.501 | NA | NA | + | NA | 0.148 | NA | NA | 4 | -28.299 | 65.254 | 12.051 | 0.000 |
| 26 | -12.771 | 0.013 | NA | NA | + | 0.123 | NA | NA | 4 | -28.342 | 65.340 | 12.137 | 0.000 |
| 61 | -12.361 | NA | NA | + | + | 0.120 | 0.005 | NA | 6 | -26.231 | 65.887 | 12.684 | 0.000 |
| 58 | -12.388 | 0.007 | NA | NA | + | 0.119 | 0.007 | NA | 5 | -27.446 | 65.892 | 12.689 | 0.000 |
| 30 | -13.036 | -0.011 | NA | + | + | 0.132 | NA | NA | 6 | -26.237 | 65.898 | 12.695 | 0.000 |
| 71 | -1.340 | NA | + | + | NA | NA | NA | -0.018 | 6 | -26.286 | 65.995 | 12.792 | 0.000 |
| 85 | -13.859 | NA | NA | + | NA | 0.150 | NA | -0.019 | 5 | -27.825 | 66.651 | 13.448 | 0.000 |
| 8 | -1.933 | -0.003 | + | + | NA | NA | NA | NA | 6 | -26.663 | 66.750 | 13.547 | 0.000 |
| 53 | -14.031 | NA | NA | + | NA | 0.142 | 0.005 | NA | 5 | -27.938 | 66.876 | 13.673 | 0.000 |
| 126 | -10.347 | -0.011 | NA | + | + | 0.118 | 0.008 | -0.042 | 8 | -24.194 | 66.913 | 13.710 | 0.000 |
| 22 | -14.744 | -0.010 | NA | + | NA | 0.154 | NA | NA | 5 | -28.073 | 67.145 | 13.942 | 0.000 |
| 49 | -14.334 | NA | NA | NA | NA | 0.152 | 0.009 | NA | 3 | -30.709 | 67.805 | 14.602 | 0.000 |
| 62 | -12.579 | -0.012 | NA | + | + | 0.126 | 0.005 | NA | 7 | -25.942 | 67.815 | 14.612 | 0.000 |
| 4 | -2.416 | 0.018 | + | NA | NA | NA | NA | NA | 4 | -29.726 | 68.108 | 14.905 | 0.000 |
| 117 | -13.295 | NA | NA | + | NA | 0.143 | 0.005 | -0.020 | 6 | -27.390 | 68.205 | 15.002 | 0.000 |
| 113 | -13.184 | NA | NA | NA | NA | 0.150 | 0.009 | -0.024 | 4 | -29.822 | 68.299 | 15.096 | 0.000 |
| 72 | -1.289 | -0.001 | + | + | NA | NA | NA | -0.018 | 7 | -26.280 | 68.492 | 15.289 | 0.000 |
| 86 | -14.072 | -0.009 | NA | + | NA | 0.155 | NA | -0.018 | 6 | -27.638 | 68.701 | 15.498 | 0.000 |
| 54 | -14.166 | -0.012 | NA | + | NA | 0.147 | 0.005 | NA | 6 | -27.653 | 68.729 | 15.526 | 0.000 |
| 50 | -14.349 | 0.011 | NA | NA | NA | 0.148 | 0.007 | NA | 4 | -30.208 | 69.072 | 15.869 | 0.000 |
| 68 | -1.595 | 0.018 | + | NA | NA | NA | NA | -0.023 | 5 | -29.065 | 69.129 | 15.926 | 0.000 |
| 18 | -15.058 | 0.017 | NA | NA | NA | 0.156 | NA | NA | 3 | -31.475 | 69.338 | 16.135 | 0.000 |
| 114 | -13.420 | 0.008 | NA | NA | NA | 0.148 | 0.007 | -0.022 | 5 | -29.507 | 70.013 | 16.810 | 0.000 |
| 118 | -13.372 | -0.011 | NA | + | NA | 0.147 | 0.006 | -0.020 | 7 | -27.122 | 70.174 | 16.971 | 0.000 |
| 3 | -1.482 | NA | + | NA | NA | NA | NA | NA | 3 | -31.907 | 70.201 | 16.998 | 0.000 |
| 82 | -14.417 | 0.015 | NA | NA | NA | 0.159 | NA | -0.020 | 4 | -30.854 | 70.364 | 17.161 | 0.000 |
| 17 | -14.749 | NA | NA | NA | NA | 0.161 | NA | NA | 2 | -33.302 | 70.794 | 17.591 | 0.000 |
| 67 | -0.628 | NA | + | NA | NA | NA | NA | -0.023 | 4 | -31.176 | 71.007 | 17.804 | 0.000 |
| 81 | -13.903 | NA | NA | NA | NA | 0.163 | NA | -0.024 | 3 | -32.378 | 71.142 | 17.940 | 0.000 |
| 105 | -0.505 | NA | NA | NA | + | NA | 0.011 | -0.036 | 4 | -31.345 | 71.346 | 18.143 | 0.000 |
| 41 | -1.648 | NA | NA | NA | + | NA | 0.009 | NA | 3 | -33.158 | 72.703 | 19.500 | 0.000 |
| 13 | -1.510 | NA | NA | + | + | NA | NA | NA | 4 | -32.096 | 72.847 | 19.644 | 0.000 |
| 77 | -0.437 | NA | NA | + | + | NA | NA | -0.029 | 5 | -31.036 | 73.071 | 19.868 | 0.000 |
| 106 | -0.708 | 0.004 | NA | NA | + | NA | 0.010 | -0.035 | 5 | -31.240 | 73.480 | 20.277 | 0.000 |
| 73 | -0.166 | NA | NA | NA | + | NA | NA | -0.035 | 3 | -33.601 | 73.590 | 20.387 | 0.000 |
| 109 | -0.623 | NA | NA | + | + | NA | 0.009 | -0.032 | 6 | -30.257 | 73.939 | 20.736 | 0.000 |
| 45 | -1.706 | NA | NA | + | + | NA | 0.006 | NA | 5 | -31.502 | 74.005 | 20.802 | 0.000 |
| 74 | -0.694 | 0.011 | NA | NA | + | NA | NA | -0.034 | 4 | -32.847 | 74.349 | 21.146 | 0.000 |
| 42 | -1.875 | 0.006 | NA | NA | + | NA | 0.008 | NA | 4 | -32.942 | 74.540 | 21.337 | 0.000 |
| 9 | -1.344 | NA | NA | NA | + | NA | NA | NA | 2 | -35.312 | 74.814 | 21.611 | 0.000 |
| 14 | -1.307 | -0.005 | NA | + | + | NA | NA | NA | 5 | -31.998 | 74.996 | 21.793 | 0.000 |
| 10 | -1.840 | 0.011 | NA | NA | + | NA | NA | NA | 3 | -34.396 | 75.178 | 21.975 | 0.000 |
| 78 | -0.308 | -0.004 | NA | + | + | NA | NA | -0.028 | 6 | -30.987 | 75.398 | 22.195 | 0.000 |
| 46 | -1.459 | -0.007 | NA | + | + | NA | 0.007 | NA | 6 | -31.347 | 76.118 | 22.915 | 0.000 |
| 110 | -0.410 | -0.006 | NA | + | + | NA | 0.009 | -0.031 | 7 | -30.142 | 76.216 | 23.013 | 0.000 |
| 5 | -1.099 | NA | NA | + | NA | NA | NA | NA | 3 | -36.862 | 80.112 | 26.909 | 0.000 |
| 37 | -1.279 | NA | NA | + | NA | NA | 0.005 | NA | 4 | -36.043 | 80.742 | 27.539 | 0.000 |
| 6 | -0.813 | -0.007 | NA | + | NA | NA | NA | NA | 4 | -36.659 | 81.974 | 28.771 | 0.000 |
| 69 | -0.916 | NA | NA | + | NA | NA | NA | -0.004 | 4 | -36.826 | 82.307 | 29.104 | 0.000 |
| 38 | -0.947 | -0.009 | NA | + | NA | NA | 0.006 | NA | 5 | -35.742 | 82.484 | 29.282 | 0.000 |
| 101 | -1.029 | NA | NA | + | NA | NA | 0.006 | -0.006 | 5 | -35.969 | 82.937 | 29.734 | 0.000 |
| 70 | -0.651 | -0.007 | NA | + | NA | NA | NA | -0.004 | 5 | -36.629 | 84.258 | 31.055 | 0.000 |
| 102 | -0.690 | -0.009 | NA | + | NA | NA | 0.007 | -0.006 | 6 | -35.666 | 84.756 | 31.553 | 0.000 |
| 33 | -0.455 | NA | NA | NA | NA | NA | 0.009 | NA | 2 | -41.032 | 86.254 | 33.051 | 0.000 |
| 34 | -0.749 | 0.008 | NA | NA | NA | NA | 0.007 | NA | 3 | -40.596 | 87.579 | 34.376 | 0.000 |
| 97 | -0.014 | NA | NA | NA | NA | NA | 0.009 | -0.011 | 3 | -40.751 | 87.890 | 34.687 | 0.000 |
| 98 | -0.336 | 0.007 | NA | NA | NA | NA | 0.008 | -0.010 | 4 | -40.386 | 89.428 | 36.225 | 0.000 |
| 2 | -0.669 | 0.015 | NA | NA | NA | NA | NA | NA | 2 | -43.536 | 91.262 | 38.059 | 0.000 |
| 66 | -0.397 | 0.015 | NA | NA | NA | NA | NA | -0.007 | 3 | -43.442 | 93.270 | 40.067 | 0.000 |
| 1 | 0.061 | NA | NA | NA | NA | NA | NA | NA | 1 | -45.717 | 93.497 | 40.294 | 0.000 |
| 65 | 0.367 | NA | NA | NA | NA | NA | NA | -0.008 | 2 | -45.584 | 95.358 | 42.155 | 0.000 |


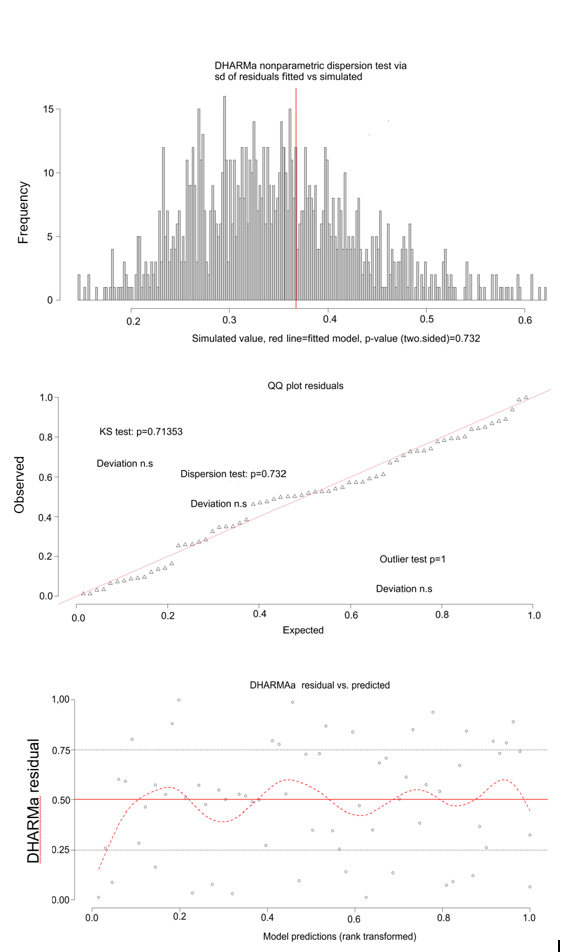


Figure S1: Test of dispersion (top most), QQ plot for normality of residuals (middle) and residuals against the fitted value (bottom) for the best model used for the model averaging on factors affecting the habitat selection of smooth-coated otter in Suklaphanta National Park, Nepal


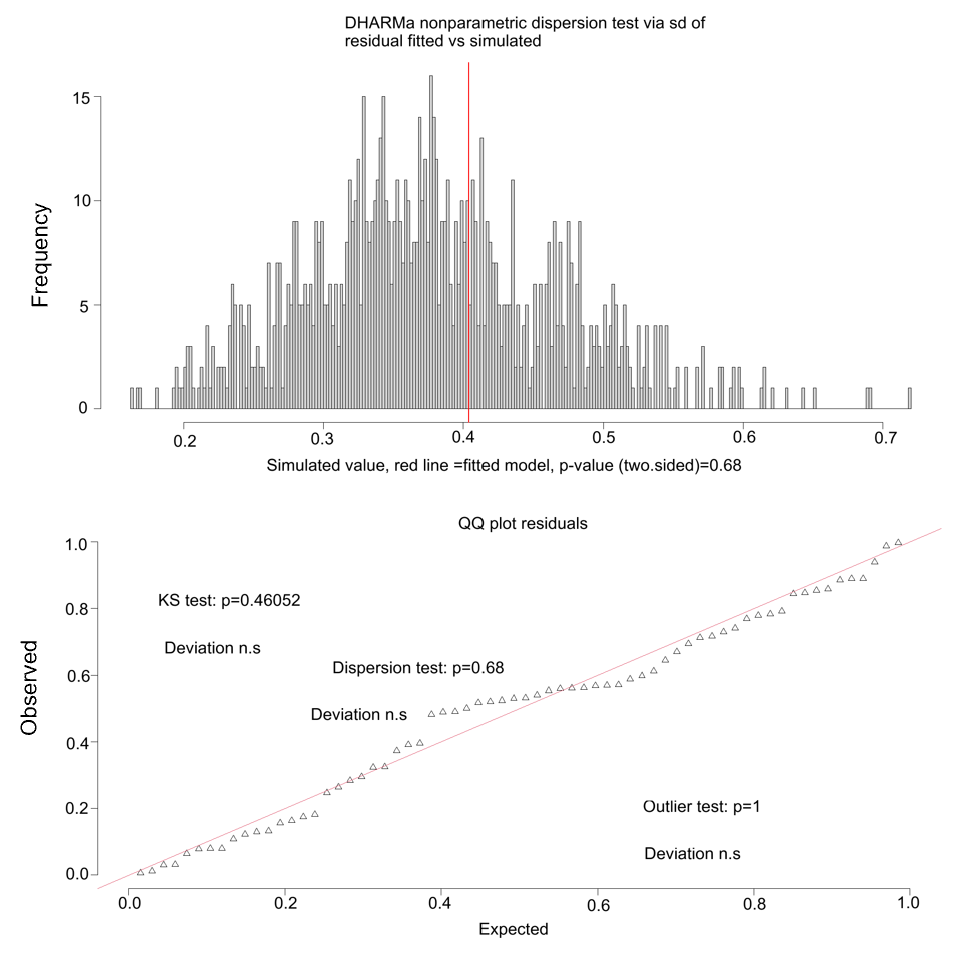


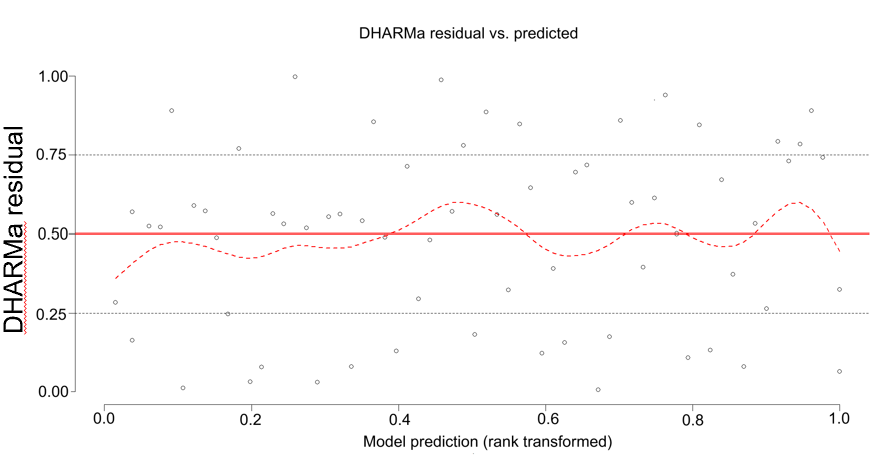


Figure S2: Test of dispersion (top most), QQ plot for normality of residuals (middle) and residuals against the fitted value (bottom) for the second best model used forthe model averaging on factors affecting the habitat selection of smooth-coated otter in Suklaphanta National Park, Nepal


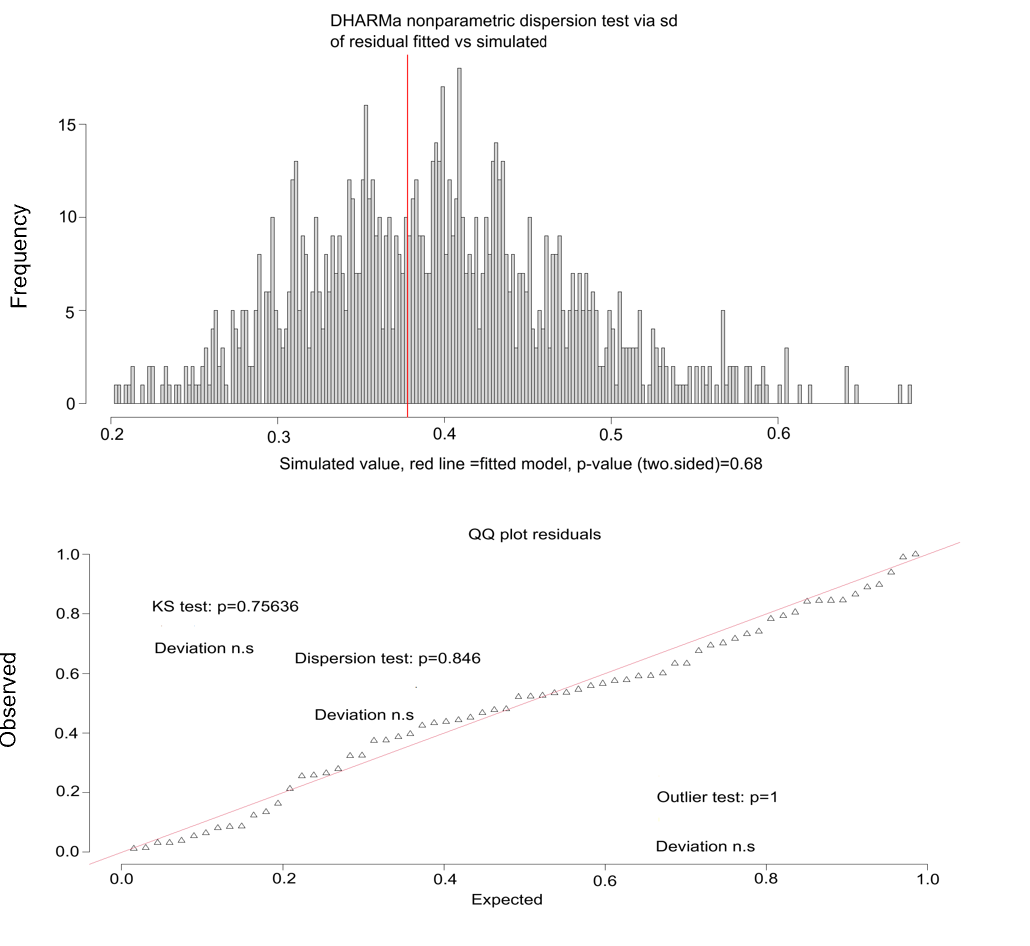


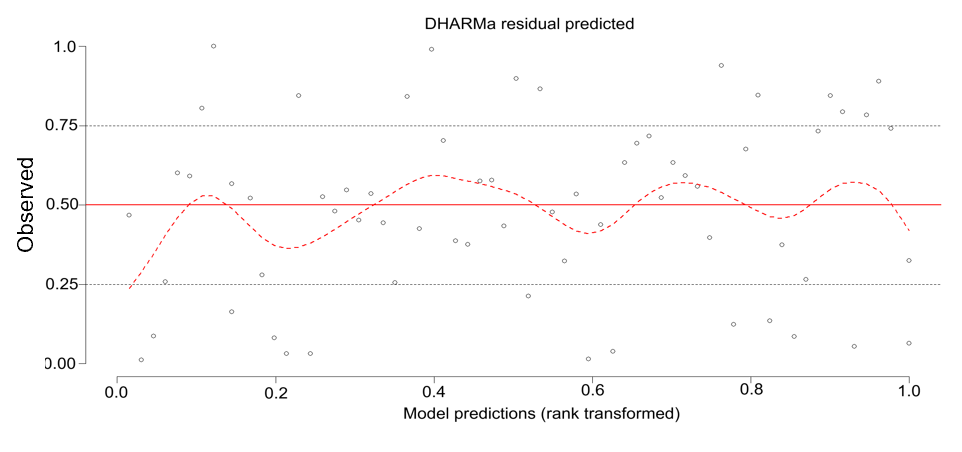


Figure S3: Test of dispersion (top most), QQ plot for normality of residuals (middle) and residuals against the fitted value (bottom) for the third best model used for the model averaging on factors affecting habitat selection of smooth-coated otter in Suklaphanta National Park, Nepal
